# Modulating neuroplasticity for chronic pain relief: noninvasive neuromodulation as a promising approach

**DOI:** 10.1038/s12276-025-01409-0

**Published:** 2025-03-03

**Authors:** Nishani Jayanika Jayathilake, Tien Thuy Phan, Jeongsook Kim, Kyu Pil Lee, Joo Min Park

**Affiliations:** 1 Department of Physiology, College of Veterinary Medicine, Chungnam National University, Daejeon, Republic of Korea; 2 Center for Cognition and Sociality, Institute for Basic Science, Daejeon, Republic of Korea; 3 Sungkyunkwan University, Suwon, Republic of Korea

**Keywords:** Chronic pain, Biological therapy

## Abstract

Chronic neuropathic pain is a debilitating neuroplastic disorder that notably impacts the quality of life of millions of people worldwide. This complex condition, encompassing various manifestations, such as sciatica, diabetic neuropathy and postherpetic neuralgia, arises from nerve damage or malfunctions in pain processing pathways and involves various biological, physiological and psychological processes. Maladaptive neuroplasticity, known as central sensitization, plays a critical role in the persistence of chronic neuropathic pain. Current treatments for neuropathic pain include pharmacological interventions (for example, antidepressants and anticonvulsants), invasive procedures (for example, deep brain stimulation) and physical therapies. However, these approaches often have limitations and potential side effects. In light of these challenges, interest in noninvasive neuromodulation techniques as alternatives or complementary treatments for neuropathic pain is increasing. These methods aim to induce analgesia while reversing maladaptive plastic changes, offering potential advantages over conventional pharmacological practices and invasive methods. Recent technological advancements have spurred the exploration of noninvasive neuromodulation therapies, such as repetitive transcranial magnetic stimulation, transcranial direct current stimulation and transcranial ultrasound stimulation, as well as innovative transformations of invasive techniques into noninvasive methods at both the preclinical and clinical levels. Here this review aims to critically examine the mechanisms of maladaptive neuroplasticity in chronic neuropathic pain and evaluate the efficacy of noninvasive neuromodulation techniques in pain relief. By focusing on optimizing these techniques, we can better assess their short-term and long-term effects, refine treatment variables and ultimately improve the quality of neuropathic pain management.

## Noninvasive neuromodulation offers hope for chronic pain relief

Chronic neuropathic pain is a serious health issue affecting many people worldwide. It results from nerve damage and involves complex changes in the nervous system, making it difficult to treat. Researchers are exploring new ways to manage this pain by focusing on neuroplasticity. This study reviews noninvasive techniques that aim to modulate neuroplasticity to alleviate chronic pain. The authors examined various noninvasive methods, such as transcranial magnetic stimulation, transcranial direct current stimulation and ultrasound, which target specific brain areas involved in pain processing. These techniques can alter neural activity without surgery, offering a safer alternative to traditional treatments. The findings suggest that these methods can reduce pain by influencing neural circuits and promoting beneficial changes in the brain. However, the effects are often temporary, highlighting the need for further research to develop long-lasting solutions.

This summary was initially drafted using artificial intelligence, then revised and fact-checked by the author.

## Introduction

Pain is defined by the International Association for the Study of Pain as “an unpleasant sensory and emotional experience associated with actual or potential tissue damage, or described in terms of such damage”^1^. Within the broader field of pain research, chronic neuropathic pain has garnered notable attention because of its high prevalence, complex mechanisms and profound impact on quality of life. Neuropathic pain arises from various conditions, including spinal cord injuries, peripheral nerve damage and diseases such as multiple sclerosis and diabetes^2^. According to the World Health Organization^3^, chronic pain affects approximately one in five people in the general population. Therefore, the World Health Organization designates chronic pain, including neuropathic pain, as a global health priority, given its widespread impact and the challenges associated with its management. Chronic neuropathic pain is increasingly recognized as a neuroplasticity disorder caused by excitatory and inhibitory imbalances in pain processing pathways. Its severity depends on numerous biological, physiological and psychological factors^4^. A key aspect of neuropathic pain is central sensitization, a form of maladaptive neuroplasticity that amplifies pain signaling in the central nervous system (CNS). Importantly, modulating this unhelpful neuroplasticity can potentially alleviate persistent pain symptoms and restore normal functioning, promoting adaptive changes^5,6^.

Researchers have employed various in vivo pain models, including models of peripheral nerve injury, spinal cord injury and chemotherapy-induced neuropathy, to study the mechanisms related to central sensitization in patients with neuropathic pain^7^. These models have provided valuable insights into the molecular and cellular changes underlying neuropathic pain, such as altered ion channel expression, increased synaptic efficacy, and changes in descending pain modulation pathways. Despite the availability of various therapeutic modalities, the treatment of chronic neuropathic pain remains challenging. Current treatment approaches include the following: (1) pharmacological interventions such as anti-epileptic drugs (for example, gabapentin and pregabalin), antidepressants (for example, duloxetine and amitriptyline) and opioid/opioid-like analgesic agents; (2) invasive procedures such as spinal cord stimulation, dorsal root ganglion (DRG) stimulation and deep brain stimulation; (3) physical therapies such as physical rehabilitation, transcutaneous electrical nerve stimulation and acupuncture; and (4) psychological interventions such as cognitive behavioral therapy and mindfulness-based stress reduction^8,9^. However, these treatments often have limitations. Pharmacological approaches can lead to side effects such as drowsiness, dizziness and potential addiction. Invasive procedures carry risks of infection, bleeding and device-related complications. Moreover, the efficacy of these treatments can vary and often diminishes over time.

As a result of these challenges, interest in noninvasive neuromodulation techniques as alternative or complementary treatments for neuropathic pain is increasing. These methods aim to modulate neural activity in pain-related brain regions without the need for surgical intervention, potentially offering a safer and more accessible approach to pain management. Researchers are exploring various noninvasive neuromodulation techniques to regulate neural plasticity and alleviate chronic neuropathic pain. These techniques include repetitive transcranial magnetic stimulation (rTMS), transcranial direct current stimulation (tDCS), transcranial alternating current stimulation (tACS), transcranial random noise stimulation (tRNS), transcranial ultrasound stimulation (tUS) and reduced impedance noninvasive cortical electrostimulation (RINCE)^10^. These modalities focus on modulating pain processing networks within the brain, including areas such as the dorsal horn of the spinal cord, dorsal anterior cingulate cortex (ACC), primary somatosensory cortex (S1), insular cortex, periaqueductal gray (PAG), thalamus and brainstem reticular formation^11^.

The primary advantage of these noninvasive techniques is their ability to target specific brain regions without the need for surgery, thus reducing the risk of complications associated with invasive procedures. Additionally, these methods have potential for repeated applications, which may be crucial for managing chronic conditions such as neuropathic pain^12^. However, while current noninvasive neuromodulation techniques have shown promise in pain management, their effects are often transient. Most of these methods work by temporarily modulating the excitability of pain-related neural circuits. This limitation highlights the need to develop new approaches that can induce long-lasting or even permanent changes in neural plasticity, potentially offering more durable pain relief. The relationship between noninvasive neuromodulation and neuroplasticity represents a frontier in neuroscience research, with important implications for optimizing pain management strategies. By obtaining a better understanding of how these techniques influence neural plasticity, we may be able to refine our approaches and develop more effective, long-term solutions for chronic neuropathic pain.

In this review, we discuss the underlying mechanisms by which neural plasticity is modulated for chronic neuropathic pain management and examine the latest noninvasive neuromodulation techniques related to plasticity-mediated chronic pain regulation. We critically evaluate the efficacy of these approaches in both preclinical and clinical settings, and consider their potential for inducing long-term analgesic effects through the modulation of maladaptive neuroplasticity. Using this approach, we aim to provide a comprehensive overview of the current state of noninvasive neuromodulation in neuropathic pain management and highlight promising directions for future research and clinical applications.

## Mechanisms of maladaptive neuroplasticity in chronic pain

Chronic pain is traditionally classified into nociceptive and neuropathic categories according to the integrity of the somatosensory nervous system. For nociceptive pain, prolonged or intense exposure to noxious stimuli triggers peripheral sensitization, characterized by changes in nociceptor thresholds and the upregulation of voltage-gated sodium channels. This peripheral sensitization leads to central sensitization in the dorsal horn of the spinal cord. It involves increased excitability, the recruitment of *N*-methyl-d-aspartate (NMDA) receptors and long-term changes in nociceptive circuits^13,14^. Neuropathic pain, arising from damage or dysfunction within the somatosensory nervous system, involves distinct yet overlapping mechanisms. Peripheral nerve lesions cause ectopic activity in injured nerve fibers and trigger immune cell responses, leading to inflammation at injury sites. This peripheral inflammation signals to the CNS, where immune mediators activate microglia in the dorsal horn of the spinal cord. Once activated, microglia drive persistent changes in neuronal excitability and circuit function, thereby modulating pain processing through mechanisms of disinhibition and abnormal excitation^15,16^. Prolonged microglial activation underlies a shift from protective to maladaptive neural plasticity, further reinforcing the chronic pain state.

The persistence of neuropathic pain long after the initial injury reflects fundamental changes in sensory information processing. Both nociceptive and neuropathic pain share common features of neural adaptation and plasticity that contribute to pain chronification. Importantly, these types of pain often coexist in patients with chronic pain, resulting in complex pain profiles that demand multifaceted treatment approaches. Central to this understanding is the concept of maladaptive neural plasticity: the nervous system’s ability to reorganize its structure, function and connections in response to persistent pain signals, often in ways that perpetuate rather than alleviate the pain state^17,18^. The development and persistence of chronic pain states are thus tightly linked to this neuroplasticity.

While neural plasticity is essential for normal function and adaptation, in chronic pain, it can become maladaptive, amplifying and sustaining pain signals. This maladaptive plasticity plays crucial roles in both the development and maintenance of chronic pain conditions.

Despite substantial advances in our understanding of chronic pain mechanisms, developing effective treatments remains a considerable challenge. In this review, current therapeutic approaches and potential new strategies based on the modulation of neural plasticity are discussed. By providing a comprehensive overview of maladaptive neuroplasticity in chronic pain, this review aims to stimulate further research into innovative approaches for chronic pain management and prevention. Understanding these mechanisms is crucial for developing more effective interventions that can address the root causes of chronic pain rather than merely treating the symptoms.

### Neuroplasticity mechanisms from the peripheral system to the central system during the development and maintenance of chronic pain

**Fig. 1:**
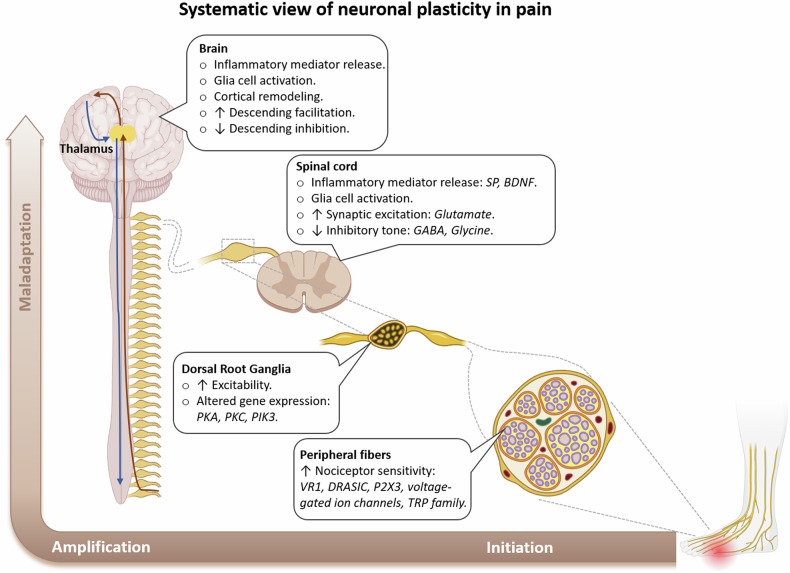
Systematic view of neuronal plasticity in chronic pain: from the peripheral nerves to the brain.

The development and maintenance of chronic pain involve complex neuroplasticity mechanisms that span from the peripheral nervous system (PNS) to the CNS (Fig. 1). This review highlights their roles in maladaptive neuroplasticity, which contribute to chronic pain.

#### Peripheral sensitization

The pain experience begins with the transmission of impulses from primary afferent fibers (Aδ and C-fibers) through the DRG. Inflammatory mediators released at sites of tissue damage, including neuropeptides such as substance P, vasoactive agents such as bradykinin and lipid-derived molecules such as prostaglandins, activate and sensitize afferent fibers by acting on nociceptor terminals^19^. The activation of excitatory receptors at afferent terminals triggers intracellular signaling pathways, including protein kinase A^20,21^, protein kinase C and phosphatidylinositol-3 kinase^22^. Activated signaling pathways result in post-translational modifications, such as phosphorylation, which alter the activation thresholds of receptors and ion channels^23^. A heat-activated cation channel such as transient receptor potential vanilloid 1 becomes more sensitive because of the decreased thresholds caused by phosphorylation^24–26^. Additionally, chronic pain syndromes may involve increased expression of voltage-gated sodium channels (for example, Na_V_1.7 and Na_V_1.8) and decreased expression of potassium channels (for example, Kv7) in neurons, which further contribute to peripheral sensitization^27,28^. An increase in sodium channel expression results in overexcitability by lowering the threshold for action potentials, thus causing pain receptors to fire in response to even minor stimuli. Conversely, decreased expression of potassium channels hinders neuronal repolarization, prolonging action potential durations and maintaining elevated excitability levels. These changes in channel expression enhance the responsiveness of nociceptors to subsequent stimuli, amplifying the perception of pain and leading to hyperalgesia.

#### Central sensitization

Central sensitization, characterized by increased neuronal excitability in the spinal cord, is a key mechanism in the development and maintenance of chronic pain^21,29^. Several factors contribute to spinal cord synaptic plasticity in chronic pain. One of them is the release of excitatory neurotransmitters such as glutamate and the reduction in the levels of inhibitory neurotransmitters such as gamma-aminobutyric acid (GABA) and glycine^30^. Another important aspect is the increased expression of pain-related receptors, particularly NMDA receptors, which leads to strengthened synaptic transmission and the prolonged activation of pain pathways^17,31^. Additionally, neuroinflammation within the spinal cord plays a crucial role in pain signaling, involving the release of proinflammatory mediators and the activation of glial cells, primarily microglia and astrocytes, which play distinct yet complementary roles in further perpetuating pain signals^25,32,33^. Microglia respond quickly to peripheral nerve injury and release proinflammatory cytokines such as interleukin-1 beta (IL-1β), tumor necrosis factor (TNF) and neurotrophins such as brain-derived neurotrophic factor (BDNF)^34^, which further accelerate neuronal excitability. Astrocytes, which are activated at a later stage and become reactive in the inflammatory process, contribute to the maintenance phase of pain sensitization by releasing glutamate, GABA, adenosine triphosphate (ATP) and other inflammatory mediators^35,36^. Both cell types thus promote the progression of neuroinflammation and accelerate pain sensitivity, further establishing central sensitization in chronic pain states. These interrelated processes collectively contribute to the establishment and maintenance of central sensitization, creating a self-reinforcing cycle of heightened pain sensitivity and persistent pain states.

#### Brain plasticity in individuals with chronic pain

Neuroplasticity mechanisms in the brain involve structural and functional alterations within various pain processing regions, including the S1, insula, ACC and prefrontal cortex^20,37,38^. These changes contribute to altered pain perception and the maintenance of chronic pain^39^. Functional magnetic resonance imaging and positron emission tomography results have revealed alterations in functional connectivity and activity within these brain regions in individuals with chronic pain^40,41^. These alterations include the increased activation and expanded representation of pain-related regions, as well as the decreased activation of regions involved in pain modulation. Increased activity in the ACC is often observed in patients with chronic pain. The ACC, which is involved in the emotional and cognitive processing of pain, typically communicates with the prefrontal cortex, which is responsible for top-down pain inhibition and cognitive control. Reduced functional connectivity between these regions can impair the ability of the brain to regulate and inhibit pain responses, leading to intensified and prolonged pain experiences. Additionally, structural changes, such as reduced synaptic connectivity and dendritic spine density, in these modulating regions further compromise pain inhibition, thereby reinforcing the persistent nature of chronic pain^14,20^.

#### Descending pain modulatory systems

The descending pain modulatory systems are triggered when pain signals reach the somatosensory cortex in the brain. The PAG and rostral ventromedial medulla (RVM) play crucial roles in the endogenous control of pain in this system. The PAG, which receives pain input from higher brain centers, processes and relays information to the RVM, which performs bidirectional pain modulation by either facilitating or inhibiting pain^42,43^. Dysfunction in these descending modulatory circuits can lead to a loss of inhibitory control and increased pain facilitation. This dysfunction also results in an imbalance between excitatory and inhibitory processes, exacerbating chronic pain^44,45^. Additionally, the impaired function of descending inhibitory pathways leads to enhanced pain perception^46^. Activation of the PAG–RVM–spinal cord pathway is associated with the development and maintenance of central sensitization^47^, and pain is modulated by inhibiting the descending pain modulatory system in neuropathic or inflammatory pain models^48,49^.

### Synaptic and structural plasticity in chronic pain

Chronic pain involves complex neuroplasticity mechanisms within both the PNS and CNS. Two key processes, synaptic plasticity and structural plasticity, play crucial roles in the development and maintenance of chronic pain states.

Synaptic plasticity refers to alterations in the synaptic structure and function in response to neural activity during chronic pain. This process primarily involves long-term potentiation (LTP) in nociceptive pathways, which amplifies and enhances pain signal transmission, and long-term depression (LTD), which potentially not only attenuates pain signals, but also influences pain signaling in a complex manner^17,21^. While LTD in excitatory pathways is well known to reduce pain transmission, LTD of inhibitory synapses within pain neural circuits plays a role in chronic pain through a disinhibitory process, leading to the reduced inhibition of nociceptive signals^50^. This disinhibition thus facilitates pain signaling paradoxically and contributes to maintaining chronic pain. The induction of LTP is primarily mediated by the activation of alpha-amino-3-hydroxy-5-methyl-4-isooxazole-propionic acid (AMPA)^51^ and NMDA receptors. AMPA receptor trafficking and phosphorylation in response to synaptic glutamate release initially contribute to increased synaptic efficacy^29^. Repetitive, high-frequency stimulation of C-fibers leads to wind-up and a progressive increase in the excitability of spinal neurons, resulting in the activation of NMDA receptors and calcium ion (Ca^2+^) influx. This Ca^2+^ influx activates various effectors, such as Ca^2+^/calmodulin-dependent protein kinase II (CaMKII), ERK and other signaling pathways. Upon activation by Ca^2+^, CaMKII undergoes autophosphorylation, which results in a persistent active state even after the removal of the Ca^2+^ signal. Similarly, activated ERK also contributes to enhancing synaptic transmission and participates in pain hypersensitivity^14,52^. Additionally, neuropeptides such as substance P and calcitonin gene-related peptide activate postsynaptic neuronal receptors, facilitating synaptic potentiation and central sensitization^25,53^.

Structural plasticity involves changes in the physical structure of neurons, including dendritic spine remodeling, axonal sprouting and synapse formation. These structural alterations are regulated primarily by activity-dependent processes and are crucial for the maintenance of chronic pain^29,53^. Key aspects of structural plasticity in chronic pain include dendritic spine remodeling and the establishment of aberrant excitatory synaptic connections in dorsal horn neurons of the spinal cord, which are associated with increased pain sensitivity. Neurotrophic factors, such as nerve growth factor (NGF) or BDNF, promote the growth and stabilization of new synapses^16,54^. BDNF facilitates the growth and stabilization of new synaptic connections, thereby enhancing synaptic transmission in pain pathways. BDNF also plays a role in restructuring dendritic spines and increasing the communication strength in spinal cord neurons, reinforcing excitatory circuits that contribute to sustained pain sensitivity^55^. Similarly, NGF supports the axonal sprouting of nociceptive fibers and the formation of new synaptic connections, maintaining and enhancing the overexcitability of the neuronal network^56^.

Understanding these plasticity mechanisms is crucial for developing targeted therapies for chronic pain. Pharmacological interventions targeting synaptic plasticity, such as NMDA receptor antagonists and modulators of neurotransmitter systems, have shown promise in preclinical and clinical studies^17,52^. For example, methadone, dextropropoxyphene and ketobemidone are all NMDA receptor antagonists that have been studied extensively in clinical trials and are effective at reducing pain sensitivity in individuals with chronic pain conditions by inhibiting excitatory synaptic transmission, further contributing to structural plasticity modulation^57^. Additionally, emerging research continues to explore ways to modulate structural plasticity, potentially by targeting the neurotrophic factors or signaling pathways involved in dendritic spine remodeling and axonal sprouting^58,59^. These approaches hold promise for developing more effective treatments for chronic pain that address the underlying neuroplastic changes rather than just managing symptoms.

### Molecular and cellular mechanisms of neuronal plasticity during chronic pain

Chronic pain is associated with substantial changes in gene expression and epigenetic modifications, which contribute to maladaptive plasticity. These changes involve complex interactions among neurons, glial cells and the immune system, leading to alterations in synaptic activity and pain processing. The activation of glial cells, particularly microglia and astrocytes, plays a crucial role in the development of chronic pain. Activated glial cells release proinflammatory cytokines (for example, IL-1β, IL-6 and TNF), contributing to neuroinflammation and modulating synaptic activity and plasticity^25,32^. Additionally, they promote the release of neurotrophic factors such as BDNF, which has been implicated in various chronic pain conditions^60^. Microglia-derived BDNF shifts the neuronal chloride gradient, enhancing nociceptive signaling^16^. This neuroimmune interaction is crucial for maintaining chronic pain as it strengthens the sensitized state of nociceptive neurons^32,61^. Epigenetic modifications, including DNA methylation and histone acetylation, also contribute notably to neuronal plasticity in chronic pain states^25^. For example, alterations of the gene that encodes glutamate decarboxylase 1, a crucial enzyme involved in GABA synthesis, are associated with decreased GABAergic inhibition, which in turn increases the excitability of pain signaling pathways^62^. Similarly, elevated levels of histone acetylation in the promoter regions of genes such as cyclooxygenase-2 and BDNF can increase their expression, resulting in increased inflammation and heightened sensitivity of pain pathways^63^. These modifications collectively contribute to the persistent sensitization of nociceptive pathways and perpetuate chronic pain.

**Fig. 2:**
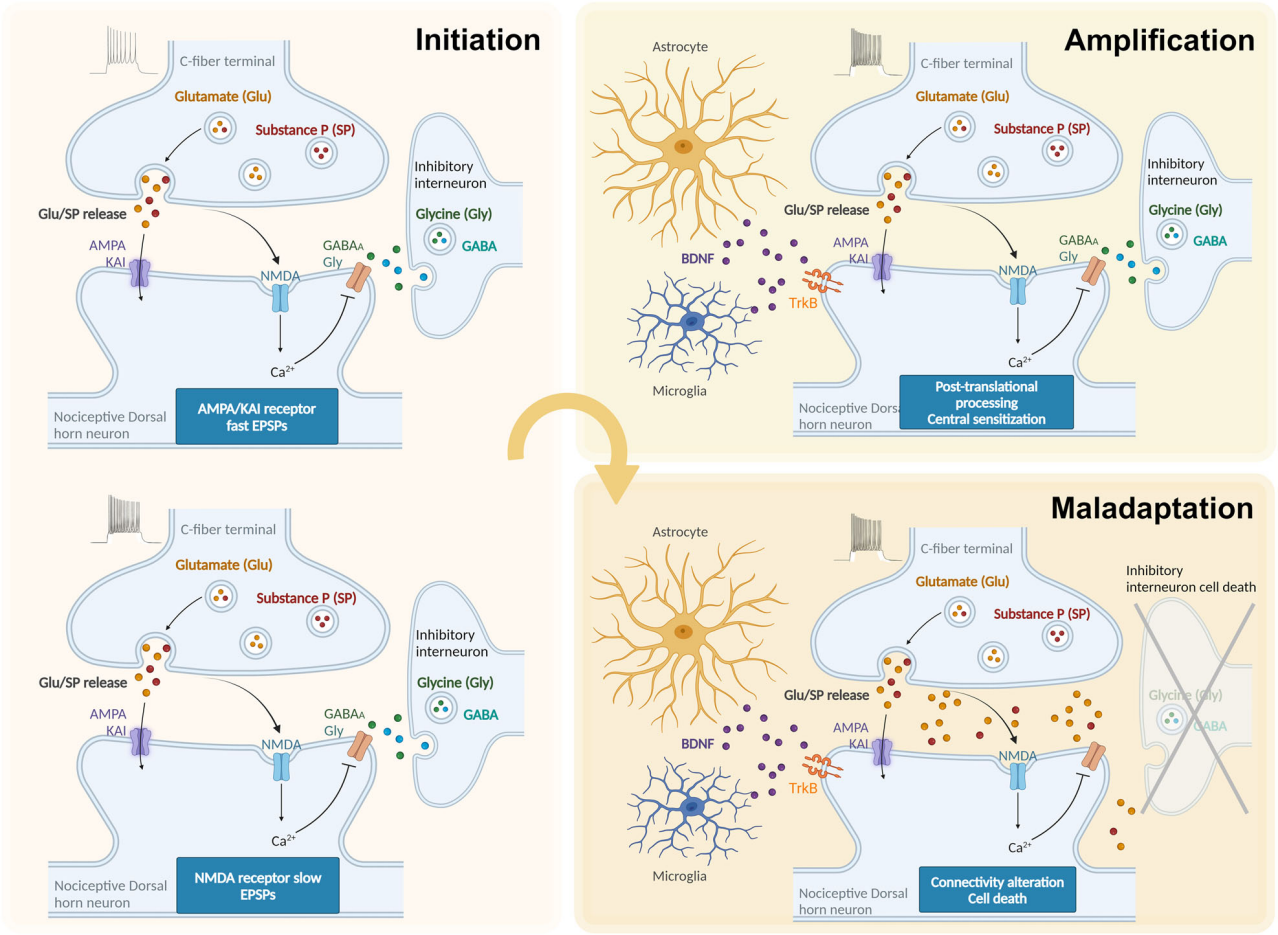
Cellular and molecular mechanisms of neuronal plasticity within the dorsal horn neurons of the spinal cord in chronic pain. Dorsal horn neurons are initially activated by fast excitatory postsynaptic potentials (EPSPs) through AMPA/kainate receptors, and this activation is further enhanced by slow EPSPs through NMDA receptors. The initiation process is mediated by intracellular kinase/phosphatase signaling and neuromodulators secreted from glial cells, which induce central sensitization. Maladaptation is driven by alterations in gene expression, the loss of inhibitory interneurons, and the formation of aberrant excitatory synaptic connections.

Prolonged stimulation from nerve injuries or persistent pain leads to maladaptive changes in the nervous system, resulting in overexcitability and altered pain processing. These changes occur within different levels of the nervous system, including peripheral nerves, the spinal cord, and the brain. The neuronal plasticity mechanisms within the system involve three main steps: initiation, amplification and maladaptation (Fig. 2). The initiation process is triggered by the release of inflammatory mediators and neurotrophic factors following inflammation or tissue damage, which activate nociceptor terminals, also called autosensitization^64^. Various receptors and ion channels have been reported to respond to these noxious stimuli, including vanilloid receptor 1 (VR1), acid-sensing ion channel (DRASIC), purinergic P2X receptor, vanilloid receptor-like protein 1 (VRL1)^17,65^, voltage-gated ion channels (for example, Na_v_1.7, Na_v_1.8 and Na_v_1.9)^26^ and ligand-gated ion channels (for example, TRPA1, TRPV and TRPM)^24,66^. After being initiated, action potentials are fired and travel to the CNS, where they reach the central nociceptor terminals in the spinal cord and result in the release of neurotransmitters. Glutamate-activated AMPA and kainate receptors are responsible for this fast excitatory synaptic transmission. This excitation is regulated by the descending input of inhibitory neurons, which release both glycine and GABA. The cumulative depolarization burst is boosted by an additional NMDA receptor current after the removal of the Mg^2+^ blockade^67^. Depolarization also activates voltage-gated calcium currents, which are mediated by calcium-activated nonselective cation channels. This increase in action potential discharge is the net effect^21^.

The amplification process subsequently occurs when the excitability of nociceptor terminals increases and generates action potentials, which are mediated by intracellular kinases activated by G protein-coupled receptors and receptor tyrosine kinases^68^. This process leads to the phosphorylation of ion channels such as the sensory neuron-specific tetrodotoxin-resistant sodium ion channel and VR1, which are associated with nociceptor sensitization^69^. Conversely, desensitizing these channels can inhibit neuropathic pain signaling. VR1 modulators, in particular, have been identified as promising candidates for pain treatment^70^. Astrocytes and microglia actively participate in this amplification process by secreting gliotransmitters and modulating neuronal excitability^52^. The maladaptation process involves alterations in growth factors, neurotransmitters and synaptic modulators in sensory neurons, contributing to system sensitization. For example, the synaptic accumulation of substance P and BDNF primarily alters the central drive evoked in dorsal horn neurons, which accelerates downstream signaling cascades, including the mitogen-activated protein kinase (MAPK) pathway, the phosphatidylinositol-3 kinase/protein kinase B (PKB) pathway, the mammalian target of rapamycin (mTOR) pathway and the nuclear factor kappa B (NF-κB) signaling pathway^17^.

In summary, the mechanisms of neuronal plasticity in chronic pain involve diverse and systemic processes, including multiple changes in neuronal and glial activity and the surrounding environment. This plasticity, while critical for the development of the nervous system, can become maladaptive in individuals with chronic pain conditions.

## Noninvasive neuromodulation techniques for neural plasticity-mediated pain modulation

**Fig. 3:**
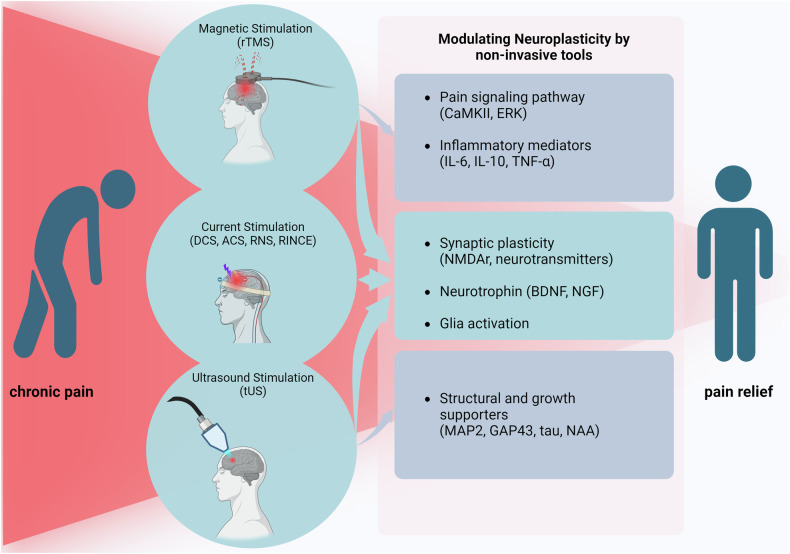
Neuronal plasticity-mediated chronic pain alleviation using noninvasive tools.

Interest in noninvasive pain treatment procedures as alternatives to pharmacological and invasive treatments is growing. Noninvasive neuromodulation techniques represent a cutting-edge approach for pain treatment (Table 1), with a focus on regulating neural plasticity (Fig. 3). Transcranial magnetic stimulation (TMS) utilizes the principles of electromagnetic induction. When electric currents pass through a primary coil, they generate a magnetic field. This magnetic flux, when flowing through the secondary coil (neural tissue), induces a secondary electrical field, causing neural stimulation^71^. A specific TMS technique, rTMS, is designed to manipulate the brain’s electrical activity by administering rapidly changing magnetic fields. rTMS has been found to alleviate several types of neuropathic pain, such as those caused by spinal cord injury, stroke, multiple sclerosis and facial pain. In many cases, it has also been shown to improve motor function^72^.

**Table 1:** Effects of noninvasive neuromodulation techniques on plasticity-mediated chronic pain.

Subject	Pain model/type of pain	Technique	Site of application	Application parameters	Effect	Plasticity related mechanism	Ref.
Rat	Peripheral nerve injury model (radial, median and ulnar nerves–right forepaw)	rTMS	Somatosensory cortex	4 s cycles of 10 Hz stimuli, 26 s interval and 7 cycles (total of 280 pulses per day, 1,680 total stimuli)	rTMS treatment immediately after injury have shown significant decrease in pain-related behavior over sham groups	rTMS treatments immediately after injury exhibited a significant alteration of CaMKII	^89^
Human	Chronic pancreatitis with visceral pain	rTMS	Secondary somatosensory cortex	Echo time of 35 ms, repetition time of 3,000 ms with 128 averages and a spectral bandwidth of 5,000 Hz	Real stimulation induced a mean decrease in pain levels of 27.2% (±24.5%), sham stimulation increases in pain levels of 1.1% (±17.1%)	Significant decrease of NAA and glutamate	^93^
Rat	Chronic constriction injury of the sciatic nerve	rTMS	Brain	1 ms duty cycle in a 1 Hz frequency, magnetic field intensity is 200 mT	Active rTMS-treated animals presented an increase in the nociceptive threshold compared with the nontreated or sham rTMS-treated rats	Significant alteration of BDNF levels, TNF, IL-10	^90^
Human	Central neuropathic pain following spinal cord injury	rTMS	Hand area of the motor cortex (M1)	Trains of 15 pulses at 10 Hz (with an intertrain interval of three seconds), total of 1,500 stimulations	rTMS over the hand area of the M1 cortex produced a transient reduction in pain intensity in acute neuropathic pain after the early phase of spinal cord injury	Altered levels of BDNF and NGF	^72^
Human	Musculoskeletal pain	rTMS	Motor cortex (M1)	Trains of 16 series of 10 s pulses at 10 Hz	Reduction in daily pain score, higher motor evoked potential in rTMS group compared with sham	Altered serum BDNF levels	^92^
Human	Cold pain	rTMS	Motor cortex (M1), dorsolateral prefrontal cortex	15 series of 10 s pulses with a frequency of 10 Hz, 50 s interval between each train, total of 1,500 pulses per session	Significant analgesic effect was observed upon stimulation	Weaker analgesic effect was observed after ketamine administration, suggesting NMDA receptor dependency	^94^
Rat	Chronic constriction injury model	rTMS	Right primary motor cortex	20 Hz, with 4 s for each sequence and a 30 s interval, 1,600 pulses	Significant reduction in brushed evoked pain score upon treatment	The expression of GFAP and the colocalization with BrdU decreased in the 20 Hz group as compared with the sham rTMS group	^138^
Rat	Osteoarthritis chronic pain model	tDCS	Brain	Current density of 0.33 mA cm^−2^	Reversed mechanical allodynia and thermal hyperalgesia	Reversed expression of NMDA receptors in PAG and strengthen the NMDA receptors-mediated antinociception	^98^
Mice	Partial sciatic nerve ligation model	tDCS	S1 in brain	0.01 to 0.1 mA with Tetrodotoxin (TTX)	Specific astrocytic activation reversed allodynia-like behavior	Initiating spine plasticity	^5^
Rat	Osteoarthritis chronic pain model	tDCS	Brain	0.5 mA (20 min per day) for 14 consecutive days	Reversed mechanical allodynia and thermal hyperalgesia	Reversed expression of NMDAR2B in spinal chord	^100^
Rat	Knee osteoarthritis chronic pain model	tDCS	Brain	0.5 mA (20 min per day) for 08 consecutive days	Reversed mechanical allodynia and thermal hyperalgesia	Protein expression of BDNF in the PAG was downregulated in the tDCS treated group	^101^
Human	Fibromyalgia	tDCS/Aerobic exercise	Primary motor cortex (M1)	Monophasic continuous current with an intensity of 2 mA for 20 min. A gradual current ramp-up and ramp-down with 30 s duration was used for stimulation	Pain intensity significantly reduced compared with sham groups	Mechanism was not investigated	^146^
Human	Side lower limb amputees	tDCS/mirror therapy	M1 cortex	20 minutes of steady-state 1.5 mA current	Pain intensity was significantly reduced after tDCS/mirror therapy	Mechanism was not investigated	^147^
Mice	Chronic constructive injury model (chronic neuropathic pain)	LIFU	ACC	Acoustic intensity of 0.95 MPa, a duty cycle of 10% and a pulse repetition frequency of 1.5 kHz, LIFU stimulation was administered for 15 min per day for 21 days	LIFU can alleviate mechanical hyperalgesia caused by chronic neuropathic pain	The expression levels of MAP2, GAP-43, and tau decreased significantly after 21 days of LIFU treatment when compared with control groups	^120^
Mice	Chronic compress injury mice model	Focused Ultrasound Stimulation (FUS)	ACC region	Pulse repetition frequency of 1.5 kHz, duty cycle of 10%, spatial-peak pulse-averaged acoustic intensity is 15,980 mW cm^−2^ and 34,982 mW cm^−2^	Alleviated mechanical neuropathic pain especially in the chronic state	Four key genes were downregulated named *Hnrnph1*, *Hnrnpd*, *Snrpb* and *Dhx16* by upon FUS stimulation. *Hnrnph1* and *Hnrnpd* are highly expressed in the brain tissue of neuropathic pain mice	^148^
Swine	Common peroneal nerve injury	LIFUS	L5 DRG	25 to 30 W for 3–4 min	Significant changes in mechanical and thermal pain behaviors and long-lasting changes in motor behavior	Mechanism was not investigated	^123^
Human	Fibromyalgia	RINCE	Deeper subcranial tissue	High-frequency carrier: greater than 10 kHz. Low-frequency component: less than 40 Hz	Significant reduction of pain VAS score	Mechanism was not investigated	^149^

Transcranial electric stimulation (tES)^73^ is one of the most commonly used noninvasive brain stimulation techniques. During tES, electrodes are applied to the scalp to deliver a current, altering neuronal excitability and modifying brain functions. A portion of the current is delivered to the inner brain, influencing specific brain regions responsible for particular behaviors. tES encompasses several techniques, including tDCS, tACS and tRNS^74^. Electrical stimulation is not limited to the brain, but is also applied to the spinal cord following spinal cord injury. Epidural electrical stimulation, peripheral nerve electrical stimulation and functional electrical stimulation have also been shown to significantly reduce pain upon application^75^. An innovative approach in this field is RINCE^76^, which facilitates deeper current penetration by decreasing the impedance of the skull and tissues. RINCE employs low-frequency current stimulation to increase precise and targeted signal transduction while increasing effectiveness^10^.

Ultrasound is another promising noninvasive neuromodulation tool that can produce remarkable biological effects when applied to the CNS and PNS^77^. The resulting biological effects can be altered using different parameters, such as frequency and amplitude^78^. These neuromodulation techniques provide a noninvasive analgesic approach in clinical and basic research settings. Their abilities to target specific neural circuits and modulate neural plasticity will offer a promising avenue for personalized pain management strategies. As research in this field progresses, these techniques will probably be refined further, leading to more effective and tailored treatments for chronic pain conditions.

### MS

Magnetic stimulation (MS), particularly TMS, has emerged as a safe and efficient noninvasive neuromodulation technique for pain management^79^. TMS generates brief and painless magnetic pulses that penetrate the skull and stimulate targeted brain areas. This stimulation can be applied in various forms, including single pulses, paired pulses with specific interstimulus intervals or rTMS, each producing distinct effects on neural excitability and sustainability. For example, continuous theta burst stimulation involves an uninterrupted burst of pulses that typically decreases excitability and pain sensation, whereas intermittent stimulation increases excitability. Monophasic TMS has a stronger short-term effect during rTMS because monophasic pulses preferentially activate a single population of neurons oriented in the same direction, allowing their effect to accumulate more effectively. In contrast, biphasic pulses might activate multiple neuron populations, including both excitatory and inhibitory groups, making the overall summation of effects less straightforward than those with monophasic pulses^80^. The efficacy of TMS in alleviating chronic pain is attributed to its ability to influence the natural electrical activity of the CNS^81^. At the cellular level, TMS induces neuroplastic changes that are crucial for pain suppression. These changes include alterations in beta-adrenergic receptor density in the cortex and striatum^82,83^, region-specific monoamine levels^84^ and the activity of the second messenger cyclic adenosine monophosphate^85^. Additionally, TMS affects the NMDA-binding site density in the hypothalamus, amygdala and parietal cortex, as well as the 5-hydroxytryptamine (5-HT, also known as serotonin) receptor density in the frontal cortex, cingulate cortex and anterior olfactory nucleus^85^. In particular, the 5-HT1A receptor is a critical subtype of serotonin receptor that binds the neurotransmitter serotonin and effectively mediates synaptic plasticity. Furthermore, TMS changes glial fibrillary acidic protein (GFAP) mRNA expression in the dentate gyrus and the cortex^86^. Hence, TMS has provided insights into effective neuroplastic changes for chronic pain suppression because of its ability to influence neurotransmitters, receptors, second messenger systems and gene expression in neural and supporting tissues and in regions that are important in pain regulation^87^.

The long-term plastic changes induced by TMS are particularly crucial for pain control^88^. Repetitive TMS has also been shown to modulate the levels of markers associated with plasticity, including BDNF, NGF, CaMKII and neurochemical modulators such as TNF and IL-10 (refs. ^72,89,90^). Studies of chronic constriction injury pain models have shown that these markers are modulated in parallel with pain alleviation by MS^90^. Notably, TMS has also been found to improve memory function impaired by neuropathic pain^90,91^. Clinical applications of TMS have shown promising results in patients with various pain conditions. When applied to the primary motor cortex (M1), TMS effectively reduces musculoskeletal pain through a top-down mechanism that mediates BDNF secretion^92^. In the case of chronic visceral pain, 1 Hz rTMS applied to the right secondary somatosensory cortex (S2) produces significant antiallodynic effects by reducing glutamate and *N*-acetyl aspartate (NAA) levels^93^. Furthermore, a human study conducted by Ciampi de Andrade et al. revealed that rTMS of the M1 region induces analgesia through NMDA- and glutamate receptor-dependent plasticity^94^. These findings collectively highlight the potential of TMS as a noninvasive neuromodulation technique for pain management. Its ability to induce neuroplastic changes in pain-related neural circuits makes it a promising tool for both clinical applications and further research into the mechanisms of pain modulation.

### DCS

A major focus on exploring the potential of direct current stimulation (DCS), particularly tDCS, in chronic pain management has been recently reported^95^. tDCS involves applying a weak electrical current to specific brain regions, with its effects varying based on the type of current (direct, alternating, pulsed or random noise), polarity (anodal or cathodal), current intensity and stimulation site^96^. The mechanism of action of tDCS in pain modulation is attributed primarily to its ability to induce long-term synaptic changes, similar to LTP and LTD.

The underlying neuroplastic mechanism of tDCS is thought to involve the calcium-dependent synaptic plasticity of glutamatergic neurons, with NMDA receptors playing a crucial role in this process^97^. Recent studies have shown that a constant direct current applied over the motor cortex and supraorbital area can induce long-term synaptic changes that alleviate chronic pain and are mediated by NMDA receptors^98,99^. This finding is further supported by research using an osteoarthritis pain model in Wistar rats, which revealed the effective top-down modulation of pain through tDCS applications. Molecular studies accompanying this research revealed changes in NMDA receptor expression in the PAG and NMDA receptor subtype 2B (NMDAR2B) expression in the spinal cord^98,100^. In addition to its effects on NMDA receptors, tDCS has also been shown to modulate pain by inhibiting BDNF and its receptor, tropomyosin receptor kinase B, which is essential for mediating the effects of BDNF on neural signaling pathways^101^. This multifaceted approach to pain modulation highlights the complex interplay between different neural pathways and signaling mechanisms in the pain response.

Minimally invasive spinal cord electrotherapy has emerged as another widely used application for alleviating pain. Multiple studies have reported considerable pain relief through the electrical stimulation of the spinal cord in both animal models and humans^102,103^. For example, Yuan et al. documented that electrical-conditioning stimulation (50 Hz, 0.2 ms for 5 min) of the spinal cord alleviates neuropathic pain in rats^102^.

Although these findings are promising, the underlying mechanisms of DCS-induced plasticity are still not fully understood. Further research is needed to comprehensively elucidate the relationship between pain relief and neural plasticity in the context of DCS. Recent advancements in DCS research have also explored the potential of combining tDCS with other neuromodulation techniques or pharmacological interventions to increase its efficacy in pain management. This approach, known as multimodal neuromodulation, may provide more comprehensive and long-lasting pain relief by targeting multiple pain pathways simultaneously. Furthermore, the development of high-definition tDCS has allowed for more focal stimulation, potentially improving the specificity and efficacy of treatment^104,105^. This advancement may lead to more personalized and targeted pain management strategies.

### ACS

tACS is a variant of tDCS that uses an oscillating sinusoidal current between electrodes instead of a constant polarity. The effects of both tACS and tDCS depend on the alignment of neurons relative to the direction of the current flow. Despite the potential of tACS for pain modulation based on oscillatory brain activity, numerous studies have suggested that its effectiveness in this area may be limited. A randomized, double-blind, controlled study investigating high-definition tACS for fibromyalgia treatment reported no significant reduction in the pain intensity or improvement in quality of life^106^. Similarly, another study in which tACS was applied to healthy participants during a pain paradigm reported no significant effect on pain modulation^107^. These findings highlight the challenges in translating the theoretical potential of tACS into practical clinical applications for pain management.

The methodology of tACS for pain control is still in its early stages and faces challenges that require further innovation and development^108^. Notably, studies explaining plasticity-based mechanisms related to pain relief by ACS are lacking, which represents a noticable gap in our understanding of its potential therapeutic effects. Recent trends in tACS research have focused on optimizing the stimulation parameters and targeting specific brain networks involved in pain processing^109^.

In conclusion, while tACS has potential as a noninvasive neuromodulation technique, its current efficacy in pain modulation remains limited. Further research is needed to elucidate the underlying mechanisms, optimize stimulation protocols and identify specific pain conditions that may benefit from the tACS intervention. As our understanding of neural oscillations and their roles in pain perception evolves, tACS may yet prove to be a valuable tool in the arsenal of pain management techniques.

### RNS

tRNS is a type of transcranial electrical stimulation that uses a low-intensity alternating current with random variations in intensity and frequency. Like tACS, tRNS employs a biphasic waveform. Biphasic waveforms are essential in tRNS because the current flow direction alternates with each cycle, which helps to avoid polarization effects at the stimulation site. The frequency spectrum typically used in tRNS studies ranges from 0.1 Hz to 640 Hz or 101 Hz to 640 Hz, with the noisy current stimulation following a Gaussian distribution with a mean of zero and a variance^110^. Few clinical studies have been conducted to evaluate the efficacy of tRNS applications. One study suggested that administering tRNS to the dorsolateral prefrontal cortex of healthy participants upon painful electrocutaneous stimulation attenuates pain perception^111^. Furthermore, tRNS has been successfully applied in clinical treatments for pain associated with multiple sclerosis^112^ and fibromyalgia^113^.

The mechanism underlying the effects of tRNS on cortical excitability is gradually being elucidated. Drug studies have revealed that the effects of tRNS on cortical excitability depend on sodium channels. Neurophysiological evidence from in vitro studies on rat neurons suggests that RNS leads to faster reopening of Na^+^ channels and affects both the peak latency and amplitude of Na^+^ currents^114^. Noisy fluctuations produced by high-frequency tRNS may increase cortical excitability by decreasing the latency and increasing the peak amplitude of Na^+^ currents entering the cell, thereby initiating membrane depolarization. The modulatory effects of tRNS are thought to be optimized at intermediate intensities. However, the optimal amount of noise in terms of the frequency range remains unclear, except for the observation that high frequencies are necessary. Nevertheless, research has shown that a wide range of frequencies also prominently impairs the modulatory effect of high-frequency tRNS on cortical excitability^51^. This finding suggests that neither the higher nor the lower subrange of the high-frequency band may be sufficient to produce the same modulation of Na^+^ channel opening and closing. While our understanding of tRNS mechanisms has improved, further research is needed to elucidate the precise mechanisms and optimal stimulation parameters fully. Additional experiments are needed to investigate the effects of various stimulation conditions and to gain a more comprehensive understanding of how tRNS modulates neural activity and influences pain perception.

### tUS

Ultrasound was first used as a diagnostic tool in medical practice and now offers a wide range of advantages^115^. Recently, ultrasound has become a noninvasive therapeutic modality for treating pain associated with several pathologies, including joint pain, neck pain and back pain^73^. Principally, mechanical waves in the range of 0.25–12 MHz^116^ penetrate either surface or deep body tissues, depending on the application. The mechanism of the therapeutic effect of ultrasound is known to be related to thermal energy conversion, cavitation, the acoustic radiation force and mechanosensitive mediators. In a clinical setting, an ultrasound transducer is placed directly on the body surface, and the energy is transferred according to an established protocol. In particular, low-intensity, low-frequency focused ultrasound has been investigated as a promising neuromodulation tool^78^ because of its ability to mediate neurochemical and functional connectivity in the brain^117^. Studies have shown that theta burst tUS (center frequency, 500 kHz; pulse duration, 20 ms; 400 pulses; spatial-peak pulse-average intensity, −33.8 W cm^−2^) applied to the deep brain in humans increases corticospinal excitability, selectively reduces GABA levels in the posterior cingulate and increases the connectivity between brain regions, suggesting that tUS mediates neuroplasticity in the human brain^118^.

Chronic pain can be alleviated by the modulation of ion channels, glutamatergic neurotransmission, cerebral blood flow, inflammation, neurotoxicity, neuronal morphology and survival, among other mechanisms, with the assistance of ultrasound^119^. However, the mechanisms are still unexplored^120^. Wang et al. reported that low-intensity focused ultrasound stimulation (LIFUS) of the ACC can effectively alleviate chronic neuropathic pain-induced allodynia by significantly decreasing the expression of neuroplasticity-related proteins. For example, microtubule-associated protein 2 (MAP2) and tau proteins support microtubule stabilization and structural integrity, whereas growth-associated protein 43 (GAP-43) is involved in axonal growth and the modulation of neurotransmitter release, and LIFUS can reduce MAP2, GAP-43, and tau levels^120^. Another study confirmed that therapeutic ultrasound application to the sciatic nerve at 1 W cm^−2^ suppressed neuropathic pain and upregulated substance P and neurokinin receptor 1 expression^121^. Interestingly, a sex-specific effect of low-intensity focused ultrasound on pain alleviation has also been observed in a common peroneal nerve injury model generated in swine. The mechanical pain threshold was increased in a sciatic nerve injury model following LIFUS of the DRG of the L5 spinal nerve, which is located in the lumbosacral region of the spinal cord, and the effect was longer in females than in males^122,123^. In summary, ultrasound has further evolved as a valuable tool for pain modulation, providing deep-tissue therapy and effective targeting.

### Other potential noninvasive techniques

Active research on the use of innovative approaches as alternative tools for invasive neuromodulation techniques such as deep brain stimulation is ongoing, and these approaches have greater future potential in pain treatment. Although these advanced approaches have yet to be widely applied to pain management, their compatibility and ongoing progress are worth noting. For example, temporal interference (TI) is one such technique for noninvasive brain stimulation that involves delivering multiple kHz-range fields with different frequencies within the range of neural activity via multiple electrodes on the scalp^124^. This technique enables focused neuronal stimulation selectively in deep tissues^125^. Mirzakhalili et al. documented the potential of indirect subthreshold activation of neurons rather than the direct activation of neurons using the lower field strength generated by temporal interference. In detail, the polarization of axonal membranes can alter synaptic transmission, leading to changes in neural spikes^126^, which represents a promising approach for influencing how pain signals are processed. Additionally, Ahtiainen et al. observed electrophysiological responses in neuron–astrocyte cocultures stimulated with TI, opening new avenues for pain modulation research^127^. Notably, the application of TI might be well tolerated, with no recorded adverse effects, and is expected to positively affect targeted brain areas related to functional connectivity in pain sensation. Transcutaneous vagus nerve stimulation (VNS), which has been approved by the United States Food and Drug Administration, is another approved approach for pain management, providing noninvasive stimulation that does not require surgeries and has minimal side effects^128^. Both transcutaneous auricular VNS (taVNS) and transcutaneous cervical VNS have emerged as noninvasive alternatives to traditional, invasive vagus nerve stimulation. Transcutaneous cervical VNS is applied to the neck surface, whereas taVNS is applied over the skin of the outer ear lobe or tragus, ultimately stimulating the auricular branch of the vagus nerve and preventing pain signals from reaching the brain^129,130^. The optimal parameters for taVNS are still being explored clinically. Current research suggests that taVNS administered for 60 s at 200% of the perceptual threshold and 10 Hz is safe and has a parameter-specific effect on heart rate^131^. The nucleus tractus solitarius in the brainstem is crucial for processing and relaying pain signals, and stimulating it through VNS has been shown to reduce pain responses, suggesting that it may underlie the antinociceptive effects of VNS^132^. Furthermore, ear stimulation via taVNS has been shown to alleviate osteoarthritic pain, postoperative pain, chronic migraines and musculoskeletal pain in the clinical setting^133–135^. Recently, photoacoustic stimulation was introduced as an alternative to traditional tUS stimulation. It was previously known as a bioimaging modality and was later innovatively transformed into a neurostimulation technology. Through this approach, neurons can be activated by ultrasound, which is generated by applying nanosecond laser pulses. In particular, photoacoustic neurostimulation has greater spatial precision without genetic modification requirements and surpasses the inherent wave diffraction limit of ultrasound while reducing the potential thermal damage to neurons and maintaining temperatures below 1 °C (ref. ^136^). Miniature ultrasound modalities such as fiber-based photoacoustic emitters have the potential to stimulate single neurons or specific subcellular structures, which support patch-clamp recordings and investigations of ion channel dynamics based on the underlying mechanism^137^. Photoacoustic stimulation is relatively new and its applications in pain management have not been widely investigated. The possibility of refining these innovative technologies as alternatives to invasive neuromodulation techniques appears promising in the future. However, the biological mechanisms underlying these techniques, as well as patient safety, efficacy and precision, should be further investigated.

### Noninvasive neuromodulation techniques for glia-mediated pain modulation

The modulation of chronic pain through noninvasive neuromodulation techniques targeting glial cells, such as microglia and astrocytes, is a relatively new and rapidly emerging field. So far, only a few studies relevant to this area have been published. According to Yang et al., GFAP/BrdU colocalization and neuropathic pain are reduced in parallel upon the application of high-frequency rTMS in a sciatic nerve injury model. GFAP is used as an astrocyte-specific marker, and BrdU is a cell proliferation marker used to identify newly formed cells. However, this observation suggests that the analgesic efficacy of rTMS is associated with the inhibition of astrocyte activation and proliferation in the spinal cord^138^.

According to Takeda et al., altering astrocytic Ca^2+^ activity through tDCS is involved in eliminating mechanical allodynia and restoring tactile sensation to normal levels. In their study, astrocytes in the S1 cortex were activated by low-intensity tDCS, together with the blockade of noxious peripheral afferent inputs aimed at the corrective remodeling of S1 circuit synapses^5^. Another study suggested that tDCS application in the rat cortical region decreases S100 calcium-binding protein B (an astrocyte-specific marker) levels, suggesting that noninvasive techniques may regulate astrocyte activity, which could be applicable to pain conditions^139^. Furthermore, the application of tDCS (0.5 mA for 20 min) has been shown to affect hippocampal astrocytes and analgesic behavior, with TNF and S100 calcium-binding protein B levels being reduced in parallel with the analgesic effect^140^. Both 2 Hz and 20 Hz electric stimulation of the sciatic nerve in a neuropathic pain model suppressed pain while decreasing astrocytic gliosis and microglial activation, along with the expression of inflammatory proteins^141^.

Microglia also dominently contribute to the modulation of pain. In particular, M1-like microglial activity is altered upon conventional electrical stimulation of the spinal cord in a rat model of chronic sciatic nerve injury^142,143^. Another study revealed that neuropathic pain conditions led to increased levels of microglia, which were reduced after repetitive intracranial electric stimulation. Even though intracranial electrical stimulation is known as an invasive tool, this study opens a new avenue for the application of noninvasive electrotherapy in both clinical and nonclinical settings. Nerve injuries cause morphological changes in microglia in higher subcortical regions that last for more than a week and are accompanied by increased expression of proinflammatory genes. Electrical stimulation was found to normalize the microglial density, soma size and activation, and was influenced by the suppression of proinflammatory cytokine levels and the induction of anti-inflammatory cytokine expression. These findings suggest that neuromodulation techniques can modulate microglial morphology and marker expression levels by interfering with inflammatory responses under neuropathic pain conditions^144^.

Few studies have shown that ultrasound can mediate chronic pain by affecting glia^138,145^. Microglial activation primarily establishes central sensitization in osteoarthritis-related pain, whereas astrocytes maintain this process during osteoarthritis progression. The activation of these glial cells triggers NF-κB, leading to the release of proinflammatory cytokines such as TNF and IL-6, which enhance nociception at the spinal level. However, low-intensity ultrasound (<1 W cm^−2^) significantly alleviated pain and suppressed glial activation, leading to a decrease in the release of proinflammatory cytokines^145^. Overall, the mechanism that involves glial cell-mediated pain alleviation via noninvasive neuromodulation techniques is not well understood. Several studies suggest that the inhibition of glial cell activation alters the balance of cytokine release, restores a normal morphology, reduces hypertrophy and hyperplasia, interferes with signal transduction pathways within glial cells and reduces neuronal overexcitability, which might affect glial activation, as potential mechanisms ameliorating pain conditions^145^. More research is needed to clarify exactly how neuromodulation influences glial cell activity and plasticity mechanisms that contribute to pain alleviation. Understanding these mechanisms could improve the effectiveness and specificity of neuromodulation therapies for chronic pain.

## Conclusions and future directions

This review summarizes recent studies investigating the underlying mechanisms of pain alleviation via noninvasive neuromodulation techniques, with a detailed focus on neuronal plasticity. Overall, noninvasive techniques have shown effectiveness in alleviating chronic pain in both human trials and chronic pain-based animal models. While relatively few studies have examined the analgesic mechanisms of these techniques, the results are promising.

MS has been the subject of comparatively more investigations, although other noninvasive neuromodulation methods have also shown appreciable potential. Further research is needed to explore patterned stimulation, optimize parameters such as the intensity and conduct comprehensive safety studies. Promising avenues for advancing research in this field include integrating these techniques with advanced neuroimaging methods, such as functional magnetic resonance imaging, and exploring the synergistic effects of combining neuromodulation tools with cognitive behavioral therapy. Moreover, delving into the involvement of glia in pain modulation opens a new area for discussion.

Despite the rapid expansion of noninvasive neuromodulation techniques, considerable knowledge gaps still remain. Further studies are needed to achieve a deeper understanding of the mechanisms involved in pain regulation. Continued research is crucial for refining the existing techniques and developing new, more effective interventions for chronic pain management.
